# Rab18 interacted with V-set and immunoglobulin domain-containing 4 (VSIG4) to involve in the apoptosis of glioma and the sensitivity to temozolomide

**DOI:** 10.1080/21655979.2021.1919012

**Published:** 2021-04-27

**Authors:** Kai Yang, Zhi Wang

**Affiliations:** Department of Neurosurgery, The First People’s Hospital of Jinzhong, Jinzhong, China

**Keywords:** Rab18, VSIG4, glioma, temozolomide, apoptosis, proliferation

## Abstract

Rab18 and V-set and immunoglobulin domain-containing 4 (VSIG4) were reportedly implicated in the malignant progression of glioma. In this study, their relationship was further explored, accompanied by the investigation into their effects on the sensitivity of temozolomide (TMZ). The proliferation and apoptosis of U87-MG and U251-MG were detected after Rab18 silencing through CCK8 assay and flow cytometry, respectively. The interaction between Rab18 and VSIG4 was predicted through database and verified by immunoprecipitation assay. The suspicion that whether the sensitivity of glioma to temozolomide was affected by the Rab18-VSIG4 interaction was explored through CCK8 assay. We observed decreased proliferation and increased apoptosis and TMZ sensitivity in U87-MG and U251-MG treated by siRNA-Rab18. Not only was the interaction predicted using database, but also it was confirmed by IP assay. Intriguingly, VSIG4 overexpression effectively reversed above biological process and TMZ sensitivity caused by Rab18 silencing. To conclude, the Rab18-VSIG4 interaction was implicated in the proliferation and apoptosis of glioma, as well as TMZ sensitivity. Targeting the interaction between Rab18 and VSIG4 may help exploit new therapies to enhance TMZ sensitivity for treating patients with glioma.

## Graphical abstract

**Figure:**
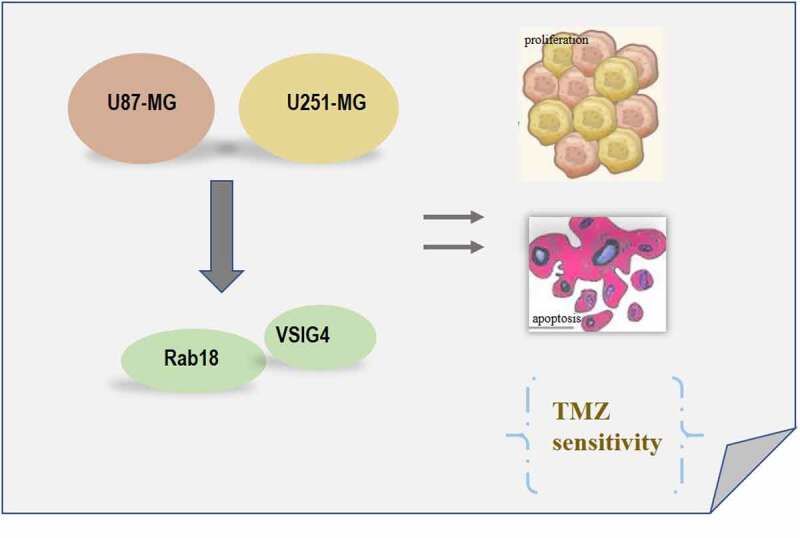

## Introduction

Glioma, which is the primary tumor with the highest degree of malignancy in the central nervous system, is mainly originated from the glial cells. Domestic and foreign research has shown that Rab18, as a member of the Rab protein family, is closely related to the progression of a variety of malignant tumors by regulating the malignant behaviors of tumors, including proliferation, migration and invasion [1–3]. Besides, it also regulated apoptosis of gastric cells and affected and mitochondrial function [4]. Clear evidence has pointed out the close relationship between Rab18 expression and prognosis and histopathology of glioma, as upregulation of Rab18 was involved in the worst outcome of glioma patients [5]. However, it remains elusive regarding the molecular mechanism by which Rab18 exerted effects on the pathophysiology of glioma.

VSIG4 is an immunoglobulin superfamily complement receptor (CRIg), also known as Z39Ig, a recently identified B7 family associated molecule [6]. VSIG4 showed abnormal expression in some cancers and it was related to the progression and prognosis [7–9]. Studies have found that VSIG4 expression presented increases in glioma tissues, which was closely associated with the prognosis of glioma [10]. In addition, it has been recently reported that VSIG4 overexpression promoted the epithelial-mesenchymal transition (EMT), invasion and migration of U87-MG glioblastoma cells, which hinted the probability that targeting VSIG4 could inhibit EMT [11]. Upregulation of CX43 by VSIG4 induction also accounted for the facilitation of glioma cells resistant to temozolomide (TMZ) [11]. TMZ is a new alkylating agent for the treatment of glioma, and by oral administration, which is the only means that TMZ gives its high bioavailability into full play in human body, it can potently penetrate the blood-brain barrier well and antagonize the malignant behaviors of glioma cells. Through searching for BioGRID4.2 database (https://thebiogrid.org/), Rab18 is predicted to physically combine with VSIG4 with combined score 0.8812. Based on these, we proposed that a possible interaction of Rab18 and VSIG4 could be involved in the progression of glioma. To explore the roles of Rab18 and VSIG4 in glioma, we silenced Rab18 or overexpressed VSIG4 to detect the proliferation and apoptosis of cells, as well as the relationship between Rab18 and VSIG. In addition, whether the Rab18-VSIG4 interaction could interfere the sensitivity of glioma cells to TMZ was also investigated.

## Methods

### Cell culture

Human glioma cells, U251-MG, U87-MG, U373-MG and T98G were purchased from American Type Culture Collection (Manassas, VA, USA) and cultured in DMEM medium containing 10% fetal bovine serum in a constant temperature incubator with 5% CO_2_, 95% humidity and 37°C. Trypsin containing 25% EDTA was used for digestion. When glioma cells adherent to the wall grew to 90% confluence, cells were collected and the cell concentration was adjusted to 1 × 10^9^/L and seeded into the 6-well plate. After 24 h, cells were used for transfection.

### Rt-qPCR

Cells of each group were collected 48 h after transfection, the mRNA of which was reverse-transcribed into cDNA according to the instructions of the reverse transcription kit (Takara). Real-time fluorescent PCR instrument Light Cycle96 was used for qRT-PCR. GAPDH was used as an internal reference. The relative expression was calculated using 2^−ΔΔCt^ method.

### Western blot

After 48 h of transfection, cells in each group were collected, the total protein of which was extracted with RIPA lysate. The protein concentration was quantified with BCA protein quantitative kit. The proteins were separated using 15%SDS-PAGE and then transferred into PVDF membrane. 5% skim milk was added to block nonspecific antigen for incubation for 2 h, followed by the incubation of primary antibodies with protein bands at 4°C overnight and secondary antibody at 37°C for 2 h. The bands were visualized with ECL reagent (Abcam, England). Image J software was used for gray analysis.

### Plasmid transfection

U87-MG or U251-MG cells were seeded into 6-well plates (3 × 10^5^ cells/well), which were transfected using siRNA-Rab18 or Ov-VSIG4 plasmids purchased from GenePharma (Shanghai, China) when the cell confluence achieved 70%. After 4 h, new medium containing 10% FBS was used for replacement of the original medium, and cell culture continued for 48 h for further experiments.

### CCK8 assay

After transfection, the cells were seeded into 96-well plates with a concentration of 2 × 10^4^/well and cultured in an incubator of 37°C with 5% CO_2_. At 0, 24, 48 and 72 h after transfection, 10 µL CCK-8 solution was added to each well. The cells were returned to the incubator for incubation for 2 h. The optical density value at 450 nm per well was measured using a microplate reader (Thermo).

### Flow cytometry

Cells in each group were collected 48 h after transfection and digested with trypsin to prepare cell suspension. Cells were resuspended with 500 µL Binding Buffer. 10 µL Annexin V-FITC and 5 µL PI were mixed and added for incubation in the dark for 30 min (Sigma-Aldrich). Novoexpress software was used to analyze the percentage of early (Annexin V+, PI-) and late apoptotic cells (Annexin V+, PI+) (Beyotime).

### Immunoprecipitation (IP) assay

After Ov-VSIG4 plasmid transfection, IP Lysis Buffer was added into U87-MG or U251-MG cell plate for cell lysis for 30 min on ice. A part of supernatant that was collected and named as Input group after centrifugation at 15,000 g at 4°C was used for western blotting analysis of Rab18 and VSIG4. 10 µL antibody anti- Rab18 was added into the other part for incubation at 4°C overnight. Next, the incubation of Protein A + G Agarose 20 µL was also conducted at 4°C for 4 h (Santa Cruz Biotechnology). Above mixture was centrifuged at 2500 rpm at 4°C to collect immunoprecipitation for western blotting analysis.

### Statistical analysis

GraphPad Prism 7.0 software was used for statistical analysis among different groups using ANOVA method, followed by Tukey’s test. P < 0.05 was considered statistically significant.

## Results

### Glioma cells expressed high RAB18

**Figure 1.**
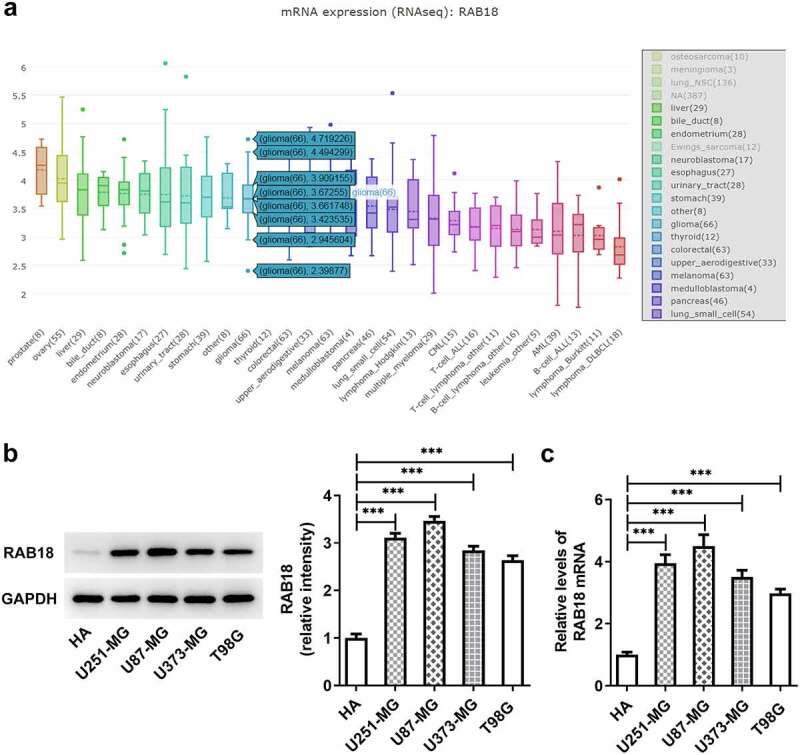
(a)the expression of RAB18 was analyzed through BioGRID4.2 database. (b–c)The protein and mRNA levels of RAB18 were detected through Western blotting and qPCR, respectively. ***p < 0.001

To identify the role of membrane trafficking protein RAB18 in glioma, we first analyzed its expression through searching for BioGRID4.2 database, and found notable increased RAB18 expression (Figure 1(a)). Additionally, the results of western blotting analysis for the expression of RAB18 indicated dramatical upregulation of protein and mRNA RAB18 levels in U251-MG, U87-MG, U373-MG and T98G cells compared with HA cells (Figure 1(b,c)), implying that RAB18 plays an essential role in glioma development.

### Silencing of RAB18 suppressed the proliferation and apoptosis of glioma cells

**Figure 2.**
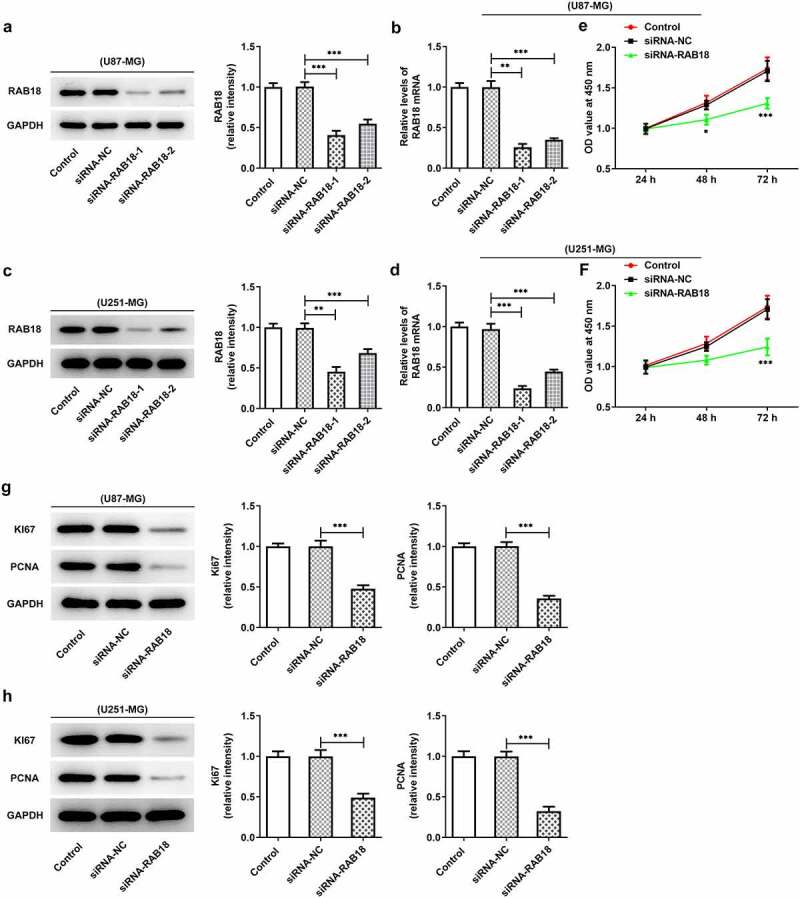
(a–d) siRNA induced the silencing of Rab18 analyzed by Western blot and qPCR. (e–f) U87-MG or U251-MG cells for Rab18 silencing expressed low proliferation. (g–h) siRNA targeting Rab18 induced a decrease of Ki67 and PCNA. **p < 0.01, ***p < 0.001

**Figure 3.**
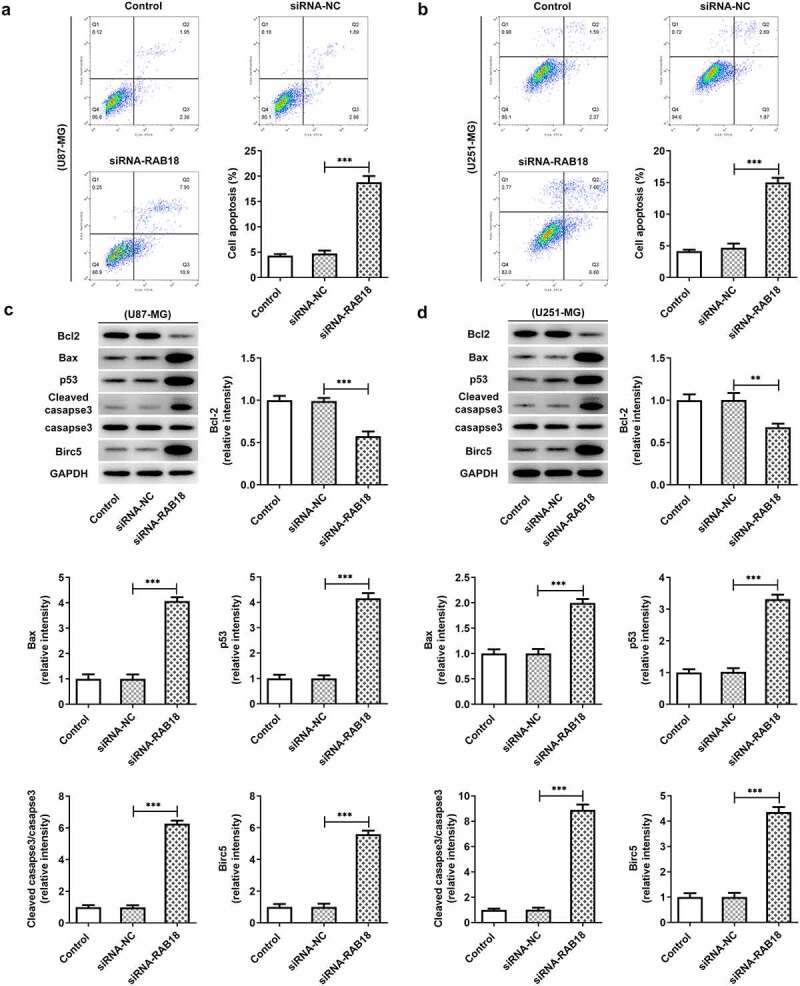
(a–b) Increased apoptosis levels were induced upon RAB18 silencing. (c–d) RAB18 silencing altered the expression of Bcl-2, Bax and cleaved caspase3. **p < 0.01, ***p < 0.001

To further investigate the role of RAB18 in glioma, we used siRNA to interfere with RAB18 expression in U251-MG and U87-MG cells. As shown in Figure 2(a–d), siRNA led to significant downregulation of RAB18 in both U87-MG cells and U251-MG cells. Silencing of RAB18 significantly induced a decrease in cell proliferation compared with siRNA-NC group (Figure 2(e–f)). What’s more, the expression of Ki67 and PCNA was also markedly reduced in U87-MG and U251-MG cells silenced by RAB18 (Figure 2(g–h)). To further dissect the role of RAB18 in apoptosis, we detected the apoptosis levels through Flow cytometry and the expression of apoptosis-related proteins by Western blotting. Intriguingly, a consequent increase in the cell apoptosis was observed upon Rab 18 silencing (Figure 3(a,b)), which was probably due to the regulation of Bcl-2, Bax, and cleaved caspase3 by Rab 18 silencing (Figure 3(c,d)).

### Rab18 silencing enhanced the sensitivity of glioma cells to TMZ

**Figure 4.**
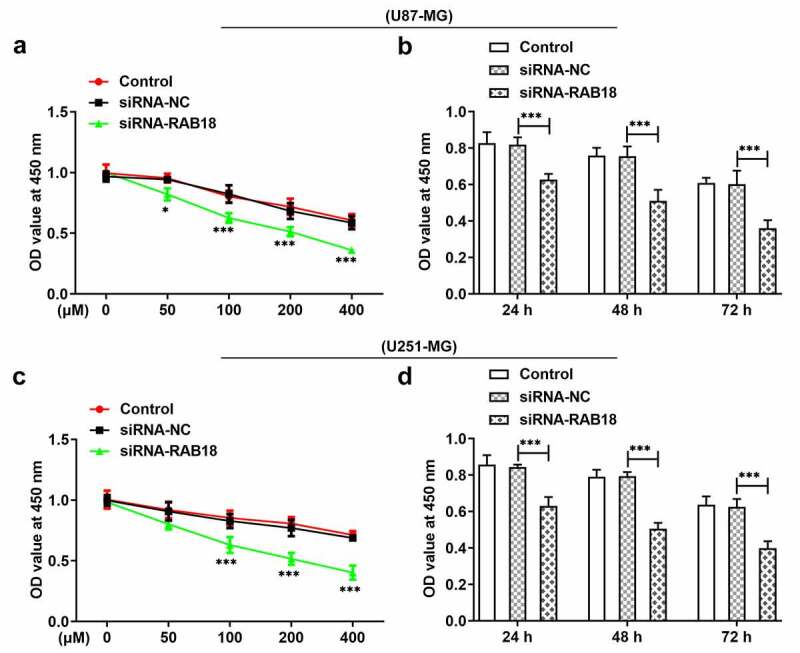
(a) The analysis of cell viability through CCk8 assay when U87-MG cells was subjected to the treatment of 0, 50, 100, 200 or 400 µM TMZ for 24 h. (b) U87-MG cells silenced for Rab18 were treated with TMZ for 24 h or 48 h. (c) The analysis of cell viability through CCk8 assay when U251-MG cells was subjected to the treatment of 0, 50, 100, 200 or 400 µM TMZ for 24 h. (b) U251-MG cells silenced for Rab18 were treated with TMZ for 24 h or 48 h. ***p < 0.001

When U87-MG or U251-MG cells silenced for Ra18 were treated with 0, 50, 100, 200 or 400 µM TMZ for 24 h, respectively, the cell viability presented dose-dependent decrease in both U87-MG and U251-MG cells (Figure 4(a,c)). RAB18 silencing induced low cell viability as compared to siRNA-NC group. Then, we treated cells with TMZ of 100 µM for 24 h, 48 h or 72 h, respectively. The results further confirmed that Rab18 silencing could enhance TMZ sensitivity of glioma cells in time-dependent manner (Figure 4(b,d)).

### Rab18 interacted with VSIG4

**Figure 5.**
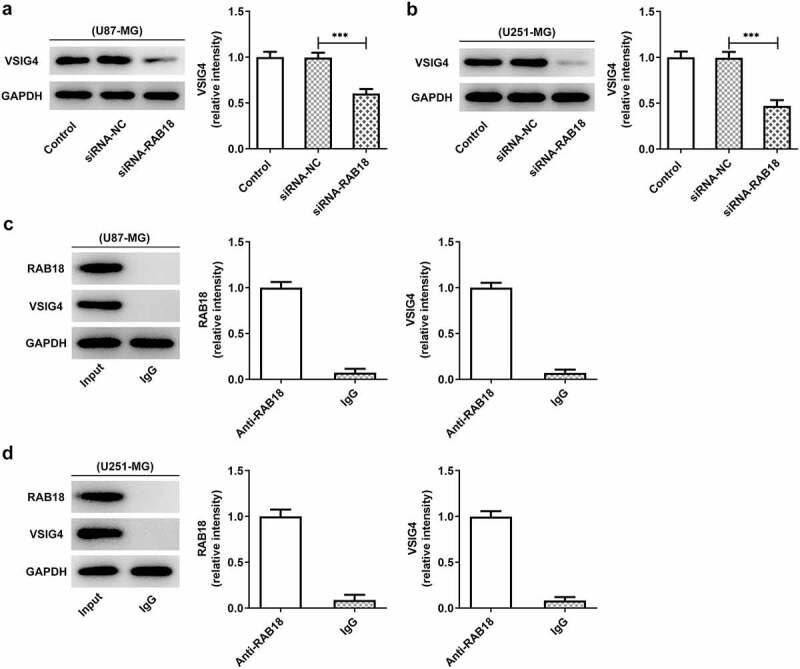
(a–b) The western blot analysis of VSIG4 was performed in U87-MG and U251-MG cells upon Rab18 silencing. (c–d) IP assay was performed to investigate the interaction of RAB18 and VSIG4. ***p < 0.001

There could exist a physical combination between Rab18 and VSIG4 through BioGRID4.2 database. We, therefore, wondered whether Rab18 affected the expression of VSIG4. We found that the induction of Rab18 silencing by siRNA not only markedly reduced the level of VSIG4 in U87-MG cells, but also reduced that in U251-MG cells (Figure 5(a–b)). We next performed IP assay to detect whether RAB18 interacted with VSIG4. Western blot analysis of the precipitated proteins showed that the protein bands of RAB18 and VSIG4 were markedly observed through using an antibody anti-RAB18 in both U87-MG and U251-MG cells (Figure 5(c–d)).

### VSIG4 overexpression interfered with the effects of Rab18 silencing on proliferation

**Figure 6.**
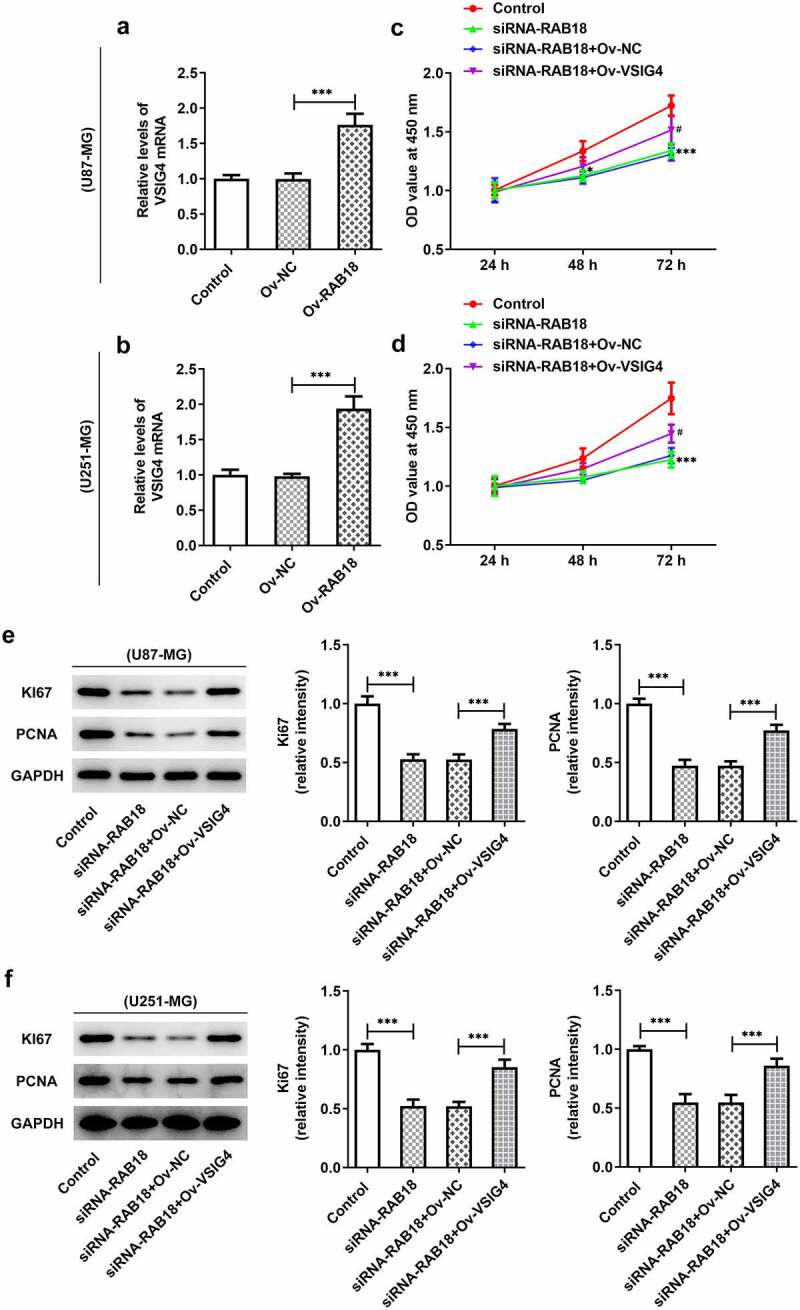
(a–b) The induction of VSIG4 overexpression elevated the VSIG4 mRNA levels both U87-MG and U251-MG cells. (c–f) VSIG4 overexpression rescued the inhibitory effects of Rab18 silencing on proliferation and proliferation markers. ***p < 0.001

The discovery of the interaction between Rab18 and VSIG4 might indicate that the complex could mediate the aformentioned functions of VSIG4. We performed VSIG4 overexpression assay and analyzed the transfection efficacy through qPCR analysis of VSIG4 mRNA in U87-MG cells and U251-MG cells (Figure 6(a–b)). The results showed that the levels of VSIG4 mRNA were effectively elevated after VSIG4 overexpression. VSIG4 overexpression was able to rescue the inhibitory effects of Rab18 silencing on proliferation of both cell lines (Figure 6(c–d)). Similar effects were also found in the expression of proliferation markers, Ki67 and PCNA (Figure 6(e–f)).

### VSIG4 overexpression rescued the influences of Rab18 silencing for apoptosis

**Figure 7.**
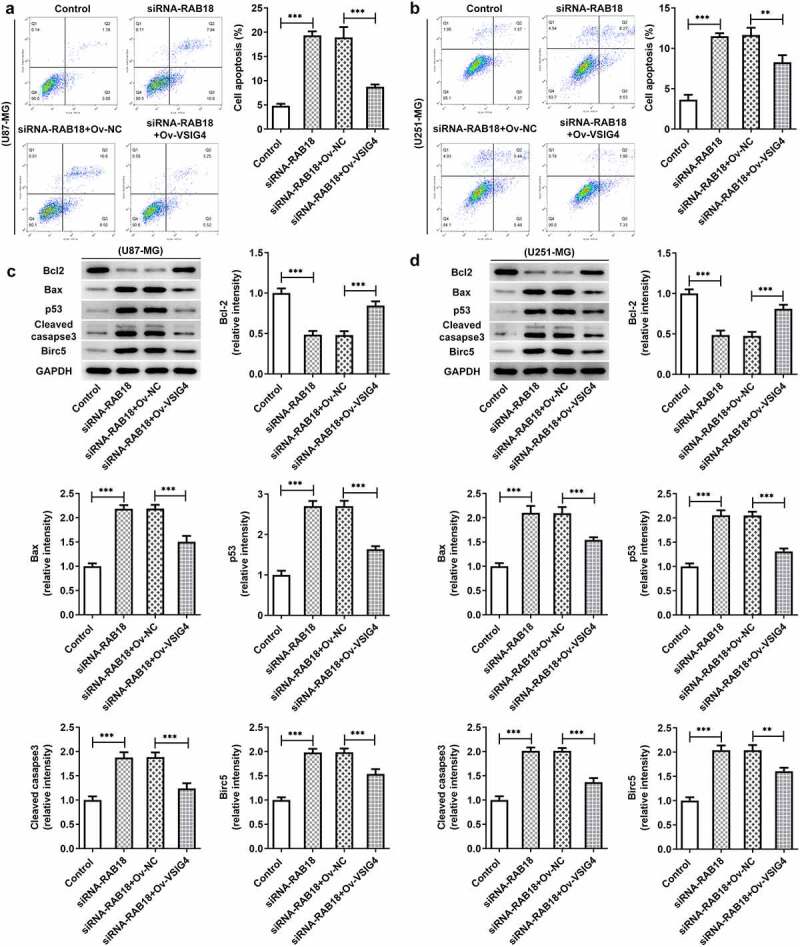
(a–b) VSIG4 overexpression led to a reverse in the effects of Rab18 silencing on apoptosis. (c–d) VSIG4 overexpression changed the expression of apoptosis-related proteins altered by Rab18 silencing. **p < 0.01, ***p < 0.001

Next, we wondered whether the interaction between Rab18 and VSIG4 was implicated in the regulation of apoptosis. The apoptosis levels and the apoptotic proteins of cells were analyzed in U87-MG cells and U251-MG cells silenced by Rab18 or overexpressed by VSIG4. The apoptosis levels through Flow cytometry analysis showed an increase in cells silenced by Rab18 than that in control group, which were restored after VSIG4 overexpression (Figure 7(a,b)). To further investigate how cell apoptosis levels were affected, we detected the expression of apoptosis-related proteins including Bcl-2, Bax, p53 and cleaved caspase3. As expected, VSIG4 overexpression marklyed rescued the effects of Rab18 on the expression of these proteins in both U87-MG and U251-MG cells (Figure 7(c,d)).

VSIG4 overexpression interfered the effects of RAB18 on drug sensitivity to TMZ.

**Figure 8.**
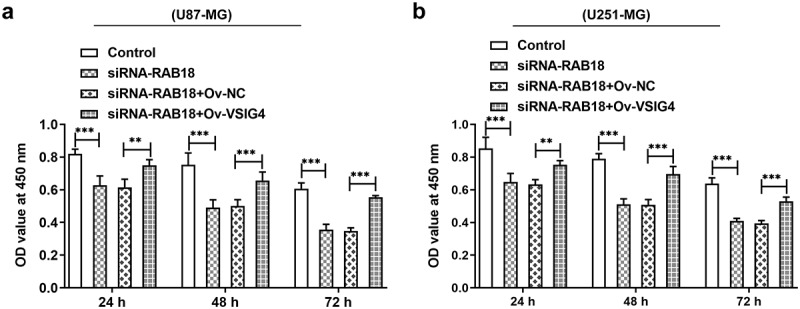
(a–b) The analysis of cell viability in U87-MG and U251-MG cells. **p < 0.01, ***p < 0.001

To further determine whether VSIG4-RAB18 affected TMZ sensitivity of glioma, CCK8 assay was performed. The observation that RAB18 silencing prominently enhanced cell sensitivity to TMZ led us to studying whether VSIG4 mediated this characteristic of TMZ. CCK8 assay showed that VSIG4 overexpression appeared to reverse the effects of RAB18 silencing on cell viability of both U87-MG and U251-MG cells (Figure 8(a–b)).

## Discussion

Rab18 has been reported to regulate cell proliferation and apoptosis in some cancers, such as hepatocellular carcinoma, gastric cancer, and head and neck squamous cell carcinoma [2,4,12,13]. Our study found that Rab18 silencing markedly suppressed cell proliferation and the expression of Ki67 and PCNA while promoted cell apoptosis in U87-MG and U251-MG cell lines. The roles of Rab18 in proliferation and apoptosis have been widely investigated in some cancers. Current studies reported that knockdown of Rab18 inhibited the proliferation of breast cancer cells while its upregulation promoted the proliferation of hepatoma cells [5,13].

The putative interaction between Rab18 and VSIG4 predicted by BioGRID4.2 database was confirmed by IP assay as significant protein levels of VSIG4 were observed in immunoprecipitation put down by the mixture of anti-Rab18 antibody and Protein A + G Agarose. These results demonstrated that Rab18 exerted inhibitory effects on proliferation and promoting effects on apoptosis possibly through interacting with VSIG4. Furthermore, VSIG4 overexpression caused significant reverse on the aforementioned effects of Rab18 silencing, suggesting that VSIG4 was engaged in mediating the function of Rab18. Our study first confirmed that there existed an interaction between Rab18 and VSIG4 in glioma cells. A study found that Rab18 interacted with transmembrane glycoprotein 5T4 and controled its expression and surface presentation [14]. VSIG4 was reported to interact with the proximal membrane adaptor protein, which further quenched Nlrp3 and IL-1β transcription through JAK2-STAT3-A20 axis [15]. However, how Rab18 interacts with VSIG4 and how this interaction mediates the signal transduction of proliferation and apoptosis are still unclarified and requires further investigation. Some research found that the regulatory role of Rab18 in regulating proliferation and drug resistance was related to the involvement of STAT3 signaling or the changes of mitochondrial functions [4,12]. Furthermore, VSIG4 was implicated in mediating the JAK2-STAT3 pathway and affected the reprogram of mitochondrial pyruvate metabolism [16,17]. These characteristics of Rab18 or VSIG4 in STAT3 signaling or mitochondrial functions may be ascribed to their interaction with VSIG4, which needs to be further explored.

All in all, these findings suggest that targeting the interaction of Rab18 and VSIG4 could contribute to marked efficacy in glioma while provides novel sights for understanding the pathogenesis of glioma and developing new therapeutical methods.

## Conclusion

We provide evidence that Rab18 plays a role in proliferation and apoptosis of glioma cells, as well as the sensitivity for TMZ through interacting with VSIG4. The study lay a crucial foundation for the development of drugs targeting Rab18 or VSIG4, and provide a novel sight for understanding of the pathophysiology of glioma.

## Data Availability

The datasets used during the current study are available from the corresponding author and the first author on reasonable request.

## Highlights

1. Rab18 silencing suppressed glioma cell proliferation and apoptosis, as well as enhancing its temozolomide sensitivity.
2. Rab18 interacted with VSIG4.
3. The interaction of Rab18 and VSIG4 affected temozolomide sensitivity of glioma cells.
